# Cancer prevention: past challenges and future directions

**DOI:** 10.1186/s41021-025-00326-y

**Published:** 2025-02-27

**Authors:** HeeKyung Seong, Runa Izutsu, Mitsuhiko Osaki, Futoshi Okada

**Affiliations:** 1 Division of Experimental Pathology, Tottori University Faculty of Medicine, 86 Nishicho, Yonago, 683-8503 Japan; 2 Chromosome Engineering Research Center, Tottori University, Yonago, 683-8503 Japan

**Keywords:** Chemoprevention, Carcinogenesis, Endogenous factors, Exogeneous factors, Oxidative stress, Medical intervention

## Abstract

Almost 70 years have passed since the molecular mechanism of carcinogenesis was hypothesized to involve multiple gene mutations. More than 1,000 cancer-related genes, including oncogenes and tumor suppressor genes, accelerate carcinogenesis by altering molecular functions and gene expression through mutations and epigenetic changes and have been shown to cause multistep carcinogenesis in several organ cancers. The elucidation of cancer-related gene abnormalities has led to the development of molecular-targeted therapies that focus on driver molecules, known as precision medicine, in addition to conventional treatments such as surgery, radiotherapy, and chemotherapy. Now that the mechanism of cancer development has been largely elucidated, options for cancer treatment and its outcomes have improved, and cancer research is moving to the next stage: cancer prevention. Cancer prevention using chemicals was first proposed approximately 50 years ago. It is the concept of stabilizing, arresting, or reverting precancerous lesions to normal tissues using synthetic vitamin A analogs (retinoids). Cancer chemoprevention is now considered to consist of three elements: “primary prevention,” which prevents the development of tumors and prevents benign tumors converting into more malignant ones; “secondary prevention,” which aims for early detection through cancer screening and treatment; and “tertiary prevention,” which reduces the risk of recurrence and extends the time until death from cancer through treatment. Consequently, there is no clear boundary between the prevention and treatment strategies. Therefore, chemoprevention targets the entire process, from normal cells to precancerous lesions, malignant progression of tumors, and death by cancer. Basic and clinical research has revealed that cancer prevention is influenced by race, regional, and national differences, as well as individual differences such as genetic factors, environmental factors, and lifestyle habits. This review provides an overview of the progress made in cancer prevention and summarizes future directions.

## Introduction

According to the International Agency for Research on Cancer (IARC), cancer is the leading cause of death in individuals aged < 85 years and the second leading cause of death worldwide after cardiovascular disease with 20 million new cases and approximately 10 million cancer deaths expected in 2022 [1, 2]. The risk of cancer is increasing globally owing to aging and population growth, which will peak this century [3]. The World Health Organization predicts that there will be more than 35 million new cancer cases by 2050 [4]. The global population aged ≥ 80 years is expected to triple from 157 million in 2022 to 459 million in 2050 [5]. An increase in the older adult population means an increase in cancer, with an estimated 2.6 million new cancer cases (13% of all cancer cases) and 2.1 million cancer deaths (21% of all cancer deaths) among the older adult population by 2022. In contrast, 7.4 million new cancer cases and 6 million cancer-related deaths are estimated by 2050, a roughly three-fold increase compared to the numbers of 2022 [6]. The most notable change in the aging population is the shift from high-income countries to low- and middle-income countries, which is expected to increase by more than 200% compared with the estimate for 2022 [6].

However, only in the United States, the combined mortality rate for all cancer sites has been declining by more than 1.5% per year since 1991 [1, 7]. This declining is expected to be due to primary prevention through lifestyle changes such as reduced smoking, secondary prevention through early detection, and tertiary prevention through improved treatment outcomes, as well as cancer and health education. It is estimated that more than 4 million cancer deaths are prevented annually [1].

Therefore, the importance of cancer prevention has emerged to avoid the increase in cancer incidence and mortality associated with the upcoming global population growth and aging. This review outlines the past challenges and achievements to date in cancer prevention and summarizes future directions.

## Causes of cancer

Given that more than 90% of patients diagnosed with cancer are aged ≥ 50 years, increasing age is the primary cause of most cancers that arise during an individual’s lifetime [8]. Conversely, in cases of hereditary tumors or repeated occupational exposure to some carcinogens that may increase the cancer risk of offspring [9], carcinogenesis may begin before birth and may occur not only during adolescence and young adulthood but also throughout later life [10, 11]. The risk factors for developing cancer throughout an individual’s lifetime and across generations are becoming clear.

Debate over whether genetic (heritable) or environmental factors play a major role in carcinogenesis had long been ongoing. This debate was resolved by a study that combined data from 44,788 twin pairs from Swedish, Danish, and Finnish twin registries and used statistical modeling to estimate the relative importance of heritable or environmental factors in causing cancers in 11 organs (stomach, colorectum, pancreas, lung, breast, cervix uteri, corpus uteri, ovary, prostate, bladder, and leukemia) of twins affected by cancer [12]. The findings revealed that heritable factors contribute approximately 20–40% to carcinogenesis, whereas environmental factors contribute 60–80%, although the contribution varies depending on the organ cancer [12]. This indicates that environmental factors play a larger role than heritable factors in susceptibility to sporadic cancer.

Cancer risk can be broadly divided into endogenous and exogenous factors [8, 13–16] (Table 1). Endogenous factors include aging, heritable factors, chronic inflammation, immunological status, sex, and hormones as individual characteristics, as well as random mutations that occur in the genome during DNA replication [17, 18]. In contrast, exogenous (environmental) factors include industrial pollutants in the air, toxic substances and iatrogenic radiation, infectious pathogens, many of which are exposures in the occupational or home settings [8, 19]. In addition, lifestyle or behavioral factors such as diet, nutrition, alcohol consumption, smoking, sun exposure, circadian rhythm, sedentary behavior (less physical activity), intestinal microbiota, socioeconomic status, residential environment including homelessness, and mental and psychological stress are now considered exogenous factors [2, 8, 14–16, 19–22].

**Table 1:** Cancer risk factors

Endogenous factors
1. Increased life expectancy
2. Genetic (heritable) factors
3. Chronic infection
4. Immunological status
5. Sex
6. Hormones
7. Mutation and repair errors
8. Immunosuppression after organ transplantation
Exogenous (environmental or lifestyle) factors
1. Infectious diseases
2. Diet and nutrition
3. Alcohol consumption
4. Smoking
5. Radiation and UV radiation (sun exposure)
6. Occupational or home exposure
7. Circadian rhythm
8. Industrial pollutants
9. Less physical activity
10. Intestinal microbiota
11. Obesity and diabetes
12. Socioeconomic status
13. Mental and psychological stress
14. Residential environment

Primary sources are from references [8, 14, 15, 16], and [33] with some additions and modifications

Among exogenous factors, the most prominent carcinogens are infectious pathogens, which are estimated to account for 15–20% of all human cancers [23]. These include one bacterium (*Helicobacter pylori*), seven viruses (human papillomaviruses, hepatitis B virus, hepatitis C virus, Epstein–Barr virus, Kaposi sarcoma-associated herpesvirus, human T-cell lymphotropic virus type 1, and human immunodeficiency virus 1), and three parasites (*Schistosoma haematobium*, *Opisthorchis viverrini*, and *Clonorchis sinensis*) [24]. Each of these infectious agents causes at least one type of cancer, with some causing several types. These infectious pathogens include bacteria, viruses, and parasites as exogenous factors. However, a common factor in carcinogenesis is chronic inflammation that is endogenously induced after infection [23]. Carcinogenesis caused by long-term chronic inflammation has been experimentally observed in various animal species and is a common phenomenon [25]. Endogenous and exogenous factors do not exist independently but interact with each other to cause DNA mutations and changes in gene expression, promoting the proliferation of cells with damaged DNA and contributing to carcinogenesis [25–27]. Oxidative stress is a common endogenous and exogenous factor that induces DNA damage. Thus, attempts to control oxidative stress have been primarily targeted for cancer chemoprevention [25].

## Cancer chemoprevention

Cancer chemoprevention research is thought to have begun with the success of experimentally creating artificial cancer in animals. This carcinogenesis research, which corresponds to the dawn of cancer research, began when Dr. Yamagiwa and Dr. Ichikawa experimentally demonstrated for the first time the “carcinogenic stimulus theory” proposed by Dr. Virchow [28]. They succeeded in inducing cancer in approximately 30% of rabbits by applying coal tar to their ears and rubbing them every day for 70 to 660 days and published the first report of their results in German in 1915 [29] and in English in 1918 [30]. This result was the first in the world to prove that carcinogenesis is caused by chemicals, and this discovery led to the rise and development of research on environmental carcinogenesis, chemical carcinogenesis, carcinogens, mutagens, and cancer chemoprevention.

In 1953, Dr. Nording proposed that the mechanism of carcinogenesis involves the occurrence of multiple (approximately six genes) mutations [31]. Subsequently, in 1971, Dr. Knudson proposed the hypothesis that for carcinogenesis to occur, both alleles of a tumor suppressor gene must lose their function through mutations or deletions [32]. Following the multistep carcinogenesis hypothesis of the 2000s [33], it is now accepted that cancer is a DNA disease, which the accumulation of a series of genetic mutations converts normal cells into cancer cells. Before these concepts were established, Dr. Berenblum was the first to experimentally discover the potential for cancer prevention using chemical dichloroethyl sulfide, also known as mustard gas, in 1929 [34]. In 1976, Dr. Sporn proposed cancer chemoprevention using synthetic vitamin A analogs (retinoids) to slow or stop early precancerous lesions or revert precancerous cells to normal cells as cancer evolves and progresses to invasive cancer [35]. Furthermore, following the proposal of the basic concept of chemoprevention by Dr. Hong and Dr. Sporn [36], chemoprevention is currently classified into three types. Primary prevention involves preventing tumor development in healthy individuals and preventing benign tumors from becoming malignant. Secondary prevention involves the early detection of tumors through screening and treatment in healthy individuals and high-risk groups. Tertiary prevention involves reducing the risk of recurrence and extending the time to death through cancer treatment in high-risk groups, patients with cancer, and cancer survivors. Cancer chemoprevention targets the entire process from normal cells to precancerous lesions, malignant progression of tumors, and death by cancer, including those derived from foods, natural products, and pharmaceuticals (Table 2). More recently, green chemoprevention, a plant-based diet and/or plant extract-based intervention with cancer-preventive effects, was introduced by Dr. Fahey and Dr. Kensler in 2012 [37].

**Table 2:** Effects of candidate compounds for cancer prevention, preventive measures, and target cancer types

Chemopreventive compounds or measures	Expected effects	Target cancer types (including potential)
1. Antioxidants, vitamins, and trace elements		
Carotenoids	Antioxidant potential	
Carotene		
β-Carotene		Not certain, but possible oral cancer, esophageal cancer, pharynx cancer, larynx cancer, and lung cancer
Lycopene		Prostate cancer
Xanthophylls		
Lutein		Inconclusive
Retinoids		
Vitamin A	Antioxidant potential	Inconclusive
All-trans retinoic acid	Induction of cell differentiation	Acute promyelocytic leukemia
Acyclic retinoids	Retinoid X receptor α modification and cancer stem cell injury	Hepatocellular carcinoma
B vitamins (Folic acid)	Cell proliferation and regeneration	Inconclusive
Vitamin C	Antioxidant potential	Inconclusive
Vitamin D	Regulation of bone metabolism and inflammation, etc.	Breast cancer, colorectal cancer, and prostate cancer
Vitamin E	Antioxidant potential	
α-Tocopherol		Inconclusive
Tocotrienols		Inconclusive
Multivitamins	Antioxidant potential	Inconclusive
Calcium	Cell proliferation and differentiation	Inconclusive
Polyphenols	Antioxidant potential	Not certain, but possible oral cancer, lung cancer, breast cancer, colorectal cancer, and prostate cancer
Trace elements		
Selenium	Antioxidant potential	Colorectal cancer
Iron	Oxidation due to excessive accumulation	Lung cancer, upper aerodigestive cancer, pancreas cancer, colorectal cancer, bladder cancer, prostate cancer, etc.
Polysulfur	Antioxidant potential	Not certain, but possible inflammation-related carcinogenesis
2. Anti-inflammatory agents		
Non-steroidal anti-inflammatory agents		
Aspirin	Cyclooxygenase inhibition, etc.	Colorectal cancer
5-Aminosalicylates	Inhibition of leukotriene B4, inflammation, etc.	Colorectal cancer
3. Metabolic regulators		
Statins	Inhibits cholesterol synthesis	Liver cancer
Metformin	Inhibits gluconeogenesis	Liver cancer, breast cancer, and uterine cervical cancer
4. Hormone replacement agents		
Anti-estrogens		
Selective estrogen receptor modulators	Competitive inhibition of estrogen from binding to the receptor (ER)	
Tamoxifen		Breast cancer
Raloxifene		Breast cancer
Aromatase inhibitors	Inhibits the conversion of androgens to estrogens	
Anastrozole		Breast cancer
Exemestane		Breast cancer
Anti-androgens		
5α-Reductase inhibitors	Inhibits the conversion of androgens to active testosterone	
Finasteride		Prostate cancer
Dutasteride		Prostate cancer
5. Medical intervention, screening, and treatment		
Vaccines		
	HBV vaccination	Hepatocellular carcinoma
	HPV vaccination	Uterine cervical cancer
Eradication	Anti-inflammation by removing *Helicobacter pylori*	Stomach cancer
Nucleos(t)ide analogues	Inhibition of reverse transcriptase of HBV	Hepatocellular carcinoma
Direct-acting antiviral agents	Eliminates the virus by targeting HCV proteins	Hepatocellular carcinoma
Parasitic anthelmintics	Removal of inflammation-causing substances	
*Schistosoma haematobium*		Squamous cell carcinoma of the bladder
*Opisthorchis viverrini*		Cholangiocarcinoma
*Clonorchis sinensis*		Cholangiocarcinoma and pancreatic cancer
Surgery to remove precancerous lesions	Removal of tissues that progress to cancer	Uterine cervical cancer, skin cancer, and colorectal cancer
Molecular targeted therapy	Inhibition of molecular abnormalities involved in carcinogenesis and progression	
		Non-small cell lung cancer, breast cancer, stomach cancer, gastrointestinal stroma tumor, hepatocellular carcinoma, pancreatic cancer, renal cell carcinoma, colorectal cancer, prostate cancer, leukemia, lymphoma, etc.

The main source of reference [38] with some additions and modifications

## Prevention with dietary and natural antioxidants, vitamins and trace elements

Dietary antioxidants and vitamins, mainly plant-derived food components, have long been studied as potential candidates for health promotion, disease prevention, and cancer chemoprevention because of their protective effects against oxidative stress [37–39]. Generally, chemopreventive agents are administered over a long period and must meet certain requirements, such as high tolerability, minimal side effects, low cost, and flexible administration schedules [40]. Consequently, dietary compounds represent promising candidates for the prevention of cancer.

Carotenoids consist of more than 600 compounds, of which approximately 50 are present in the diet and 25 are absorbed by the body [38]. Carotenoids are classified into carotenes and xanthophylls. β-Carotene and other carotenes are referred to provitamin A because they are converted into retinol. Foods containing carotenoids have preventive effects on cancers of the mouth, lungs, pharynx, and larynx, as well as β-carotene have preventive effects against esophageal cancer [38], positioning β-carotene as a candidate for cancer prevention. Some studies have shown preventive effects in subjects with poor nutritional status; however overall there is no preventive effect against the development of cancer, and in some cases may promote carcinogenesis. Four large-scale clinical intervention trials targeting lung cancer have been conducted. (i) Physicians’ Health Study (1982): American male physicians were administered β-carotene and aspirin, β-carotene had no effect on cancer-related deaths [41]. (ii) Linxian Study (1986): Administration of β-carotene, vitamin E, and selenium to Chinese subjects was shown to have a preventive effect against all cancers, while administration of high doses of β-carotene to smokers increased the incidence of lung cancer [42]. (iii) Carotene and Retinol Efficacy Trial (CARET; 1988): β-carotene and retinol were administered to current and former smokers and asbestos-exposed Americans, but the trial was discontinued due to increased lung cancer incidence and mortality, as well as cardiovascular disease-related mortality [43]. (iv) Alpha-Tocopherol Beta Carotene Cancer Prevention Study (ATBC Study; 1994): Finnish male smokers were administered β-carotene and α-tocopherol, and β-carotene alone or in combination with α-tocopherol increased the incidence of lung cancer, prostate cancer, stomach cancer, and overall mortality [44]. Increased dietary intake or blood levels of the carotenoid lycopene are directly related to reduced prostate cancer risk [45].

Retinoids did not show any preventive effect against lung cancer in the CARET and ATBC studies, whereas administration of retinol and β-carotene administration increased the incidence of cancer [46]. All-*trans*-retinoic acid (ATRA), a type of retinoid, is used clinically as a differentiation-inducing therapy to differentiate acute promyelocytic leukemia cells into neutrophils [47]. Acyclic retinoids, synthetic vitamin A derivatives, have been shown to suppress recurrence in patients with liver cancer and are currently undergoing clinical trials as potential drugs to prevent recurrence [48]. One of the targets of action of acyclic retinoids was the nuclear receptor retinoid X receptors α [49], which is responsible for the function of retinoic acid, and the other was found to be against liver cancer stem cells expressing the oncogene N-*Myc* [50]. Acyclic retinoids are expected to be the first agent in the world capable of suppressing liver cancer and its recurrence and metastasis in patients with chronic hepatitis, liver cirrhosis, and hepatocellular carcinoma, who are at high risk for developing and recurrence of liver cancer.

Cancer prevention with folic acid appears to be optimal in a dose-dependent manner. Folic acid deficiency promotes colonic carcinogenesis, moderate doses inhibit it, and high doses promotes it [38]. Case-control studies have shown that folic acid reduces the risk of colorectal cancer after ulcerative colitis [51, 52]; however, no large-scale epidemiological studies have provided evidence for chemopreventive agents [40].

Vitamin C exists in both reduced and oxidized forms. Reduced vitamin C levels cooperates with those of glutathione and vitamin E to suppress oxidative stress [53]. Conversely, oxidized vitamin C can function as an oxidant through the Fenton reaction in the presence of transition metals [20, 54], leading to lipid peroxidation [55]. Reduced vitamin C is a promising cancer prevention agent; however, there is currently no evidence that it inhibits cancer risk, at least at the dietary level [56], and large-scale clinical trials have shown no effect on the risk of cancer development at all sites [57–59].

The activated vitamin D receptor is expressed in cancer cells and inhibits β-catenin signaling, making it a promising chemopreventive agent [60]. However, epidemiological studies have suggested differential effects on cancer risk depending on the organ [61]. Based on large-scale clinical trials [62–64], the IARC concluded that serum 25-hydroxyvitamin D3 concentrations were inversely correlated with colorectal cancer [38]. Observational studies have shown a strong association between low circulating 25-hydroxyvitamin D3 levels and a higher risk of developing breast, colorectal, and prostate cancers [65–70]. Vitamin D also appear to be related to cancer aggressiveness, with elevated serum levels being associated with arresting noninvasive prostate cancer and inhibiting its progression to invasive ones [71]. Conversely, vitamin D deficiency is associated with an increased risk of bladder cancer [72].

Vitamin E contained in foods is divided into two groups, tocopherols and tocotrienols, based on the side chain structure, and further classified into eight types in total including α, ß, γ, and δ isoforms. The majority of vitamin E in the body is α-tocopherol, which primarily scavenges peroxyl radicals and stabilizes long-chain polyunsaturated fatty acids in cell membranes [38]. Cancer chemoprevention trials with vitamin E include the ATBC Study [44] for lung cancer and the Selenium and Vitamin E Cancer Prevention Trial (SELECT) for prostate cancer; however, neither trial was successful in reducing the risk of cancer [73].

The National Institutes of Health has reported that multivitamin supplements are beneficial for cancer prevention in individuals with poor nutritional status or low antioxidant intake but have no preventive effect in individuals on a normal diet [74]. The World Cancer Research Fund concluded that multivitamin supplements are not beneficial for cancer survivors and may be harmful at high doses [56].

Large-scale observational studies have shown an inverse correlation between calcium administration and the incidence of colorectal adenoma and colorectal cancer [56, 75]. This might be due to calcium, adsorption and excretion of secondary bile acids that stimulate colonic epithelial cells, as well as their effect on the inhibition of epithelial cell proliferation and differentiation [76].

Phenols are aromatic compounds with one hydroxyl group. Compounds with two or more phenolic groups on an aromatic ring are referred to as polyphenols [77]. Plants contain over 8,000 types of polyphenols, several hundred of which are found in foods [77, 78]. Owing to the antioxidative properties of their hydroxyl groups, polyphenols have been considered potential chemopreventive agents for oxidative stress-related diseases, including cancer, inflammation, neurodegeneration, cardiovascular disease, diabetes, and infectious diseases [77]. Although there are no conclusive epidemiological studies on the preventive effects of polyphenols against carcinogenesis of the lungs, breast, colon, and prostate have been shown [78–83]. The molecular mechanisms of chemoprevention by polyphenols encompass several functions beyond their antioxidative properties. These include induction of cell cycle arrest and apoptosis, inhibition of cell proliferation, inhibition of angiogenesis, inactivation of carcinogens by the induction of detoxification enzymes, anti-inflammation, immune activation, normalization of signal transduction pathways, and both estrogenic and antiestrogenic effects [84, 85]. In addition, they control the expression of oncogenes and tumor suppressor genes. Among these, p53 gene acts on multiple molecules involved in carcinogenesis [86]. Many polyphenols not only directly induce the expression of the p53 protein, but also inhibit its degradation of p53 protein by ubiquitination through phosphorylation or acetylation, thereby extending and stabilizing its half-life [86–88]. Carcinogenesis prevention is being attempted by reactivating the function of other tumor suppressor genes for RB. Polyphenol-rich Kakadu plum juice and pomegranate juice, which reactivate RB gene function, were screened [89]. These juices have antioxidant and anti-inflammatory effects and prevent colon carcinogenesis in rats [89]. This is the first study to demonstrate that a natural beverage can reactivate RB function and may be a new candidate beverage for future cancer prevention strategies. Polyphenols, characterized by their antioxidant properties, act as pro-oxidants in vivo. In particular, the oxidation of epigallocatechin, the main component of green tea polyphenols, was observed at doses higher than those required for antioxidant activity [77].

Selenium, an essential trace element, not only serves as an antioxidant but is also the active center of the antioxidative enzyme glutathione peroxidases, which reduces oxidative stress [56, 90]. Selenium reduced the incidence [40] and mortality [91] of colorectal adenoma recurrence and colorectal carcinogenesis but did not prevent lung and prostate carcinogenesis [92, 93] or tumor recurrence or progression [90]. Increased selenium intake increases the risk of kidney cancer [94].

Iron is another active center of redox reactions that can donate electrons through its high affinity to oxygen and nitric oxide. It plays a crucial role not only in oxidative energy production but also in DNA synthesis and repair as the essential cofactor of ribonucleotide reductase. Consequently, actively dividing cells compete for iron. Iron is essential for living organisms; however, excess accumulation of iron is highly reactive with oxygen (Fenton reaction) and acts as a catalyst for oxidative stress, damaging macromolecules such as genomes, proteins, and lipids, thereby promoting carcinogenesis [95]. For instance, a large-scale clinical trial showed that patients with peripheral arterial disease in whom iron stores were reduced by phlebotomy had lower cancer-specific all-cause mortality rates [42]. Experimental studies have demonstrated that phlebotomy can prevent asbestos-induced carcinogenesis [96]. Therefore, chemoprevention using phlebotomy and iron chelators is also promising in patients with hepatitis [97] and in those at a high risk of cancer due to asbestos exposure.

Amino acids with sulfur-containing functional groups, such as thiol and cysteine ​​persulfide, produced by the reaction of cysteine with hydrogen sulfide, are referred to as polysulfur molecules. These molecules exhibit stronger antioxidant potential and nucleophilicity than single thiols or hydrogen sulfide [98, 99]. Oxidative damage caused by reactive oxygen species and nitric oxide is expected to be regulated by the presence of polysulfur. Although an increase polysulfur molecules may be a promising cancer prevention agent, the carcinogenesis linked to oxidative stress need to be reevaluated in the near future, including polysulfur.

## Prevention with pharmacological compounds

Tamoxifen and raloxifene are the only cancer-preventive drugs approved in the United States for breast cancer [100]; however, nonsteroidal anti-inflammatory drugs (NSAIDs) are considered potential next-generation candidates.

Among the NSAIDs, aspirin is a well-established cancer chemoprevention agent [38]. Aspirin is one of the oldest medicines and a multipurpose drug commonly used as an analgesic, anti-inflammatory, antipyretic, and antiplatelet agent [101, 102]. For many years, aspirin has been studied for the prevention of cardiovascular diseases [103]. However, it has also been shown to potentially reduce the risk of certain cancers, particularly colorectal cancer [104]. The carcinogenesis-preventive function of aspirin is thought to be due to its inhibition of cancer progression, such as proliferation, metastasis, and thrombosis [105]. Clinical trials conducted in western countries have demonstrated that aspirin reduces the risk of death from colorectal cancer by suppressing adenoma development [106]. In Japan, the administration of low-dose enteric-coated aspirin tablets in patients undergoing endoscopic resection for colorectal cancer prevents recurrence [107]. Interestingly, when comparing non-smokers with current or former smokers, aspirin prevented colorectal cancer recurrence only in non-smokers, but this preventive effect was lost in smokers [107]. This indicates that lifestyle habits influence aspirin’s ability to prevent recurrent colorectal cancer. Low-dose aspirin is demonstrated to be effective in preventing colorectal cancer in Asian patients with familial adenomatous polyposis (FAP). When patients with FAP who had all their colorectal adenomas removed were administered aspirin and mesalazine, a drug for treating ulcerative colitis, mesalazine tended to suppress the occurrence of adenomas compared to the placebo group, whereas aspirin reduced the number of adenomas by one-third [108]. To date, no cancer chemopreventive agents are covered by health insurance in Japan; however, low-dose aspirin is expected to be the first cancer-preventive drug to be put to practical use in patients with FAP.

## Prevention through medical interventions

Hereditary cancers are associated with specific gene mutations, such as SCLC1 in lung cancer, MLH1, MSH2, MSH6, PMS2, and EpCAM in Lynch syndrome (colorectal cancer), DPC4 in pancreatic cancer, CDKN2A and CDK4 in malignant melanoma, BRCA1 and BRCA2 in breast and ovarian cancer, and HPC1 + and TLR in prostate cancer [14]. Primary prevention interventions, such as genetic counseling for individuals at high risk of certain cancers associated with inherited genetic mutations to inform them of the psychosocial issues they may encounter, have been to reduce cancer incidence [109].

In the case of inflammation-related carcinogenesis caused by infectious agents, the recommended primary preventive interventions include vaccination and bacterial eradication. The goal is to avoid or eliminate chronic inflammation by blocking the infection. Currently, the treatment of infectious diseases related to cancer prevention includes the eradication of *Helicobacter pylori* [110], HPV vaccines [111], HBV nucleos(t)ide analogs [112], HCV direct-acting antivirals [113], and HIV antiretroviral drugs [114].

Early detection of cancer through screening and the removal of precancerous lesions is also effective in preventing cancer. The latter was examined in a randomized controlled trial known as The National Polyp Study, which was conducted in patients who underwent colonoscopic polypectomy for high-grade dysplastic adenomas [115]. The results indicated that adenoma resection reduced the incidence of colorectal cancer in the short term [116] and prevented colorectal cancer mortality by 53% in the long term [117]. Moreover, the mortality rate of colorectal cancer was reduced by 53% by polypectomy, 35% through lifestyle improvements, including dietary changes, and 12% as a result of advancement in cancer treatment, leading to a total reduction of 88% from primary to tertiary cancer prevention [118]. The successful of such cancer prevention studies is supported by the fact that there are many people who are willing to actively participate in the planned program, and by the existence of recruitment methods and participation benefits that increase motivation of participants, such as preferential treatment for the cost of testing. As one of the efforts to obtain the cooperation of such participants, a clinical trial registration database jointly operated by the National Institutes of Health and U.S. Food and Drug Administration has been established [119], and the information is publicly available, so it is important to be able to grasp the current situation and progress.

## Conclusion

Cancer chemoprevention is meaningful not only for individuals who have not yet been diagnosed with cancer, but also for cancer survivors. It is a desirable cancer prevention measure that dispels the fear of cancer development, recurrence, metastasis, and cancer death, and its benefits are easy to accept.

Chemoprevention using dietary and natural antioxidants, vitamins, and trace elements is difficult to evaluate and must be performed with caution, considering that it is confounded by multiple factors such as differences in race, country/region, and optimal dose, as well as the influence of lifestyle habits such as smoking and the occupational environment. In contrast, cancer prevention through medical interventions or treatments demonstrates clear efficacy. In addition to removing precancerous and early detected lesions, medical interventions and treatments aimed at controlling infections and preventing inflammatory responses have also been shown to be effective in cancer prevention, as they remove the causative lesions and pathogenic infectious sources themselves. To ensure cancer preventive efforts, future developments should include exploring new chemoprevention targets and preventive compounds/drugs, identifying intermediate biomarkers to evaluate the effectiveness of prevention, and utilizing individual genetic profiles [38].

Notably, the annual cancer mortality rate in the United States declined by 1.5% from 1993 to 2001 and 2.0% from 2001 to 2006 in men, as well as by 0.8% from 1994 to 2002 and 1.5% from 2002 to 2006 in women [118]. This decline is likely because all cancer prevention strategies, from primary to tertiary prevention, have been implemented [7]. By using this successful example in the United States as a model and improving and applying them to cancers in each organ, we will steadily progress toward achieving effective cancer prevention.

## Data Availability

No datasets were generated or analysed during the current study.

## Abbreviations

ATBC: Alpha-tocopherol beta-carotene cancer prevention study
CARET: Carotene and retinol efficacy trial
FAP: Familial adenomatous polyposis
IARC: International Agency for Research on Cancer
NSAIDs: Nonsteroidal anti-inflammatory drugs
